# Antidiabetic activity of *Berberis brandisiana* is possibly mediated through modulation of insulin signaling pathway, inflammatory cytokines and adipocytokines in high fat diet and streptozotocin-administered rats

**DOI:** 10.3389/fphar.2023.1085013

**Published:** 2023-04-06

**Authors:** Shumaila Mehdi, Malik Hassan Mehmood, Mobeen Ghulam Ahmed, Usman Ali Ashfaq

**Affiliations:** ^1^ Department of Pharmacology, Faculty of Pharmaceutical Sciences, Government College University, Faisalabad, Pakistan; ^2^ Department of Biotechnology and Bioinformatics, Faculty of Life Sciences, Government College University, Faisalabad, Pakistan

**Keywords:** *Berberis brandisiana* Ahrendt, chemerin, adipocytokines, insulin receptor substrate -1, A disintigrin and A metalloproteinase 17

## Abstract

Medicinal plants play a key role in protection of chronic non-communicable ailments like diabetes, hypertension and dyslipidemia. *Berberis brandisiana* Ahrendt (Berberidaceae) is traditionally used to treat diabetes, liver problems, wounds, arthritis, infections, swelling and tumors. It is also known to be enriched with multiple phytoconstituents including berbamine, berberine, quercetin, gallic acid, caffeic acid, vanillic acid, benzoic acid, chlorogenic acid, syringic acid, *p*-coumaric acid, *m*-coumaric acid and ferulic acid. The efficacy of *B. brandisiana* has not been established yet in diabetes. This study has been planned to assess the antidiabetic activity of *B. brandisiana* in high fat diet and streptozotocin (HFD/STZ)-induced diabetes using animals. Administration of aqueous methanolic extract of *B. brandisiana* (AMEBB) and berbamine (Berb) for 8 weeks caused a dose dependent marked (*p* < 0.01) rise in serum insulin and HDL levels with a significant decline (*p* < 0.01) in glucose, triglycerides, glycosylated hemoglobin (HbA1c), cholesterol, LDL, LFTs and RFTs levels when compared with only HFD/STZ-administered rats. AMEBB and Berb also modulated inflammatory biomarkers (TNF-α, IL-6) and adipocytokines (leptin, adiponectin and chemerin). AMEBB (150 mg/kg and 300 mg/kg) and Berb (80 mg/kg and 160 mg/kg) treated rats showed a marked increase (*p* < 0.001) in catalase levels (Units/mg) in pancreas (42.4 ± 0.24, 47.4 ± 0.51), (38.2 ± 0.583, 48.6 ± 1.03) and liver (52 ± 1.41, 63.2 ± 0.51), (57.2 ± 0.58, 61.6 ± 1.24) and superoxide dismutase levels (Units/mg) in pancreas (34.8 ± 1.46, 38.2 ± 0.58), (33.2 ± 0.80, 40.4 ± 1.96) and liver (31.8 ± 1.52, 36.8 ± 0.96), (30 ± 0.70, 38.4 ± 0.81),respectively while a significant (*p* < 0.01) decrease in serum melondialdehyde levels (nmol/g) in pancreas (7.34 ± 0.17, 6.22 ± 0.22), (7.34 ± 0.20, 6.34 ± 0.11) and liver (9.08 ± 0.31,8.18 ± 0.29), (9.34 ± 0.10, 8.86 ± 0.24) compared to the data of only HFD/STZ-fed rats. Histopathological studies of pancreas, liver, kidney, heart and aorta revealed restoration of normal tissue architect in AMEBB and Berb treated rats. When mRNA expressions of candidate genes were assessed, AMEBB and Berb showed upregulation of IRS-1, SIRT1, GLUT-4 and downregulation of ADAM17. These findings suggest that AMEBB and Berb possess antidiabetic activity, possibly due to its effect on oxidative stress, glucose metabolism, inflammatory biomarkers and adipocytokines levels. Further upregulation of IRS-1, SIRT1, GLUT-4 and downregulation of ADAM17, demonstrated its potential impact on glucose homeostasis, insulin resistance and chronic inflammatory markers. Thus, this study provides support to the medicinal use of *B. brandisiana* and berbamine in diabetes.

## Introduction

Diabetes and obesity are the paramount recurrent endocrine-metabolic disorders that are categorized by hyperglycemia and impaired insulin secretion and/or its action (Kiziltas et al., 2022; Mutlu et al., 2023). Loss of β-cells function and impaired insulin secretion in obesity and diabetes results in persistent hyperglycemia and dyslipidemia (Eguchi et al., 2021). Although the etiology of diabetes is complex, however, genetic proclivity combined with an unbalanced diet play an important role in its onset and progression. Its prevalence has been increased dramatically which might be the result of sedentary lifestyle and increased consumption of high-energy foods (Sankaranarayanan et al., 2018). As per Global Diabetes Alliance, there are 537 million grown-ups with diabetes. This figure is also projected to reach 643 million by 2030 (Miaffo et al., 2021) and will further increase to780 million by 2045. It has also become one of the top 10th global causes of death (Li et al., 2020). Persistent hyperglycemia in diabetes is crucial in the development and progression of diabetes related complications possibly through induction of pro-inflammatory cytokines, reactive oxygen species and adipocytokines (Nedosugova et al., 2022). Consumption of high fat diet (HFD) and streptozotocin (STZ) in animals leads to β-cells damage and impaired insulin secretion and/or function (Hong et al., 2021). Metabolic syndrome has usually been associated with inflammation which results due to stimulation of inflammatory cytokines (TNF-α and IL-6) and adipocytokines (leptin, adiponectin and chemerin) (Zorena et al., 2020; Matthews et al., 2021). Diabetes is exacerbated by overproduction of reactive oxygen species (ROS) which disrupts the insulin signaling pathway, resulting in the development of insulin resistance in diabetes. Multiple evidences support that oxidative stress and hyperglycemia activate serine kinase cascades, which has several possible targets in the insulin signaling pathway, including insulin receptor substrate (IRS) proteins family. Increased phosphorylation of IRS at specific threonine or serine sites, causes decreased phosphorylation of tyrosine, hence resulting in impaired action of insulin (Batista et al., 2021). Serine/threonine phosphorylated IRS molecules are less likely to interrelate with insulin receptor (IR) signaling. In this case, downregulation of target molecules, particularly, phosphatidylinositol 3-kinase (PI3K) is mainly involved in impaired insulin action and glucose transport. Phosphatidylinositol 4, 5-biphosphate (PIP2), an intracellular membrane substrate, is phosphorylated by PI3K to form phosphatidylinositol 3, 4, 5-triphosphate (PIP3), which recruits signaling proteins such as AKT (Saltiel, 2021). PI3K/AKT signaling is important in cellular physiology as it mediates critical cellular processes like lipid metabolism, protein synthesis and glucose homeostasis (Camaya et al., 2022). ROS is known to play a part in activation of intracellular stress kinases and inhibition of IRS-1, thus potentially influencing insulin signaling and to promote *via* GLUT-4 translocation and down streaming of AKT resulting in insulin resistance, obesity and diabetes mellitus (Li et al., 2022).

There are growing evidences that development of insulin resistance is related with an increase in the release of pro-inflammatory cytokines and adipocytokines (Menghini et al., 2013; Al-Mansoori et al., 2021). Stimulated production of TNF- α contributes in degradation of β-cell and increased activity of ADAM 17, a mediator of TNF- α production (Al-Kuraishy et al., 2021). Imbalance between antioxidants and reactive oxygen species in diabetes usually stimulates the production of ADAM17 and TNF-α. It has been reported that there is association between increased TNF-α levels in diabetes and impaired insulin signaling possibly by increasing IRS-1 serine phosphorylation. This inhibits tyrosine kinase IR activity and thus intervening signal down streaming (Lee et al., 2022).

Sirtuin-1 (SIRT1) modulates insulin signaling through IRS after IR tyrosine phosphorylation stimulated by insulin, continues to activate AKT, resulting in positive regulation of insulin secretion in pancreatic β-cells, protection from inflammation and oxidative stress and plays important roles in the metabolic pathway through modulation of insulin signaling (Lee et al., 2009). The overexpression of SIRT 1 in diabetic animal represents significantly amelioration of glucose intolerance and insulin resistance. As a result, SIRT1 is a promising therapeutic target for the treatment of insulin resistance and diabetes (Feng et al., 2021).

Despite the availability of multiple therapeutic treatment options for treatment of diabetes, the disease progression spectra are increasing day by day. The currently available treatment options are either introducing exogenous insulin or to increase the sensitivity of insulin. These therapeutic options remain unable to provide sustained glycemic control or halt the disease progression (Bhatti et al., 2022). Herbal products have recently received a lot of attention as a complementary and/or adjuvant therapies (Abdulghafoor et al., 2021; Gall et al., 2021; Ayaz et al., 2022). The use of traditional medicinal plants in the treatment of diseases including diabetes is endorsed by WHO. Diabetes has been managed by using a variety of medicinal plants including *Zingiber officinale, Allium sativum, Elephantopus scaber, Areca catechu, Elephantopus scaber, Ricinus communis* and *Ocimum sanctum* since ancient times (Aumeeruddy and Mahomoodally, 2021).

Research findings have been published by various labs on the phytochemical and pharmacological properties of Berberis species. *Berberis brandisiana* Ahrendt is member of Berberidaceae family, a dicotyledonous genus mainly woody, spiny, evergreen shrubs and flowers (Khan and Khatoon, 2007). It consists of 17 Genera and 650 species. Berberis species belonging to this family are originated from the mountainous regions in Pakistan at sea level of above 1400–3500 m and are also used both in modern system of medicines and traditionally as well. Its vernacular names are “Ishkeen and Shugloo”. It has been traditionally used in various disorders like diabetes, kidney stones, liver problems, wounds, arthritis, infections, tumors, leucorrhoea and swellings (Khan et al., 2016). Berberis species have been widely used in Ayurveda as raw materials or as ingredients for wound healing, arthritis, eye infections, hemorrhoids, piles, reducing obesity, treating dysentery and indigestion (Khan and Khatoon, 2007; Bhardwaj and Kaushik, 2012). A number of clinical and pharmacological studies on various Berberis species have been published, demonstrating their significance as medicinal plants with great therapeutic potential (Rahimi-Madiseh et al., 2017). *Berberis aristata, Berberis chitria* and *Berberis lycium* extracts have been used as a home remedy for diabetes, bleeding piles, conjunctivitis, skin diseases, ophthalmic problems, ulcers, jaundice, inflamed spleen and liver since ancient times (Khan et al., 2016). From various parts of the Berberis plants, phytochemicals including alkaloids, phenolic acids, sterols, lignins, anthocyanins, flavanoids, carotenoids, terpenoids, vitamins, lipids and proteins were isolated (Khan et al., 2016). Berberis plants were used to extract alkaloids like berberine, berbamine, baluchistanamine, thalifoline, isotetrandrine and flavonoids rich in polyphenols like caffeic acid, quercetin, meratin, rutin, chlorogenic acid and other nutrients and minerals such as β-carotene, anthocyanin, and ascorbic acid (Khan et al., 2016). Berberis plants contain two important alkaloids, berberine and berbamine (Chander et al., 2017).

Berbamine (Berb) is a bis-benzylisoquinoline alkaloid reported to be present in Berberis plants including *Berberis aristata, Berberis vulgaris, Berberis poiretil schneid, Berberis amurensis* and *Berberis brandisiana* (Wang et al., 2009; Khan et al., 2016) belongs to Berberidaceae family. Berb has long been used in clinical settings for variety of ailments due to its reported anti-inflammatory effect by inhibiting (NF-κB, ERK1/2 and JNK signaling pathways) through the activation of macrophages and neutrophils (Jia et al., 2017), anticancer (Farooqi et al., 2022), antioxidant (Sithuraj and Viswanadha, 2018), immunomodulatory (Zhang et al., 2020), hepatoprotective (Sharma et al., 2021), cardioprotective (Sun et al., 1998; Zhang et al., 2012; Han et al., 2018; Sankaranarayanan et al., 2018) and antihypercholesterolemic effects.

Considering the therapeutic potential of *B. brandisiana* in diverse health ailments and the availability of limited data on this botanical herb, this study has been designed to investigate the antidiabetic properties of *B. brandisiana* and its metabolite, berbamine with an insight into its modulatory effects on insulin signaling pathway, inflammatory cytokines and adipocytokines using HFD/STZ-administered diabetic rats. Further quantitative expression of mRNA of diabetic candidate genes like, IRS-1, GLUT-4, SIRT 1 and ADAM17 were also studied for their role on the part of protective potential of *B. brandisiana* and berbamine in diabetes.

## Materials and methods

### Chemicals

Streptozotocin (STZ) and berbamine (Berb) were purchased from Glenthem life sciences, United Kingdom. Metformin, cholesterol and formalin were sourced from Sigma-Aldrich. ELISA kits for the assessment of TNF-α, IL-6, adiponectin insulin, leptin and chemerin; (E-EL-H0109), (E-EL-H0102), (E-EL-R3034), (E-EL-H 6122), (E-EL-R 0582) and (E-0864Ra) were purchased from Elabscience, United States. In this study, all chemicals were of analytical quality and obtained from Glenthem life sciences, United Kingdom. Other ingredients including powdered milk, vegetable oil, wheat bran, fishmeal, black treacle, wheat flour and table salt were purchased from local supplier at clock tower market, Faisalabad, Pakistan. Nutrivet-V and potassium metabisulfite were purchased from local veterinary pharmacy in Faisalabad.

### Animals

Wister rats, of either sex, weighing 180–250 g were obtained from University of Veterinary Sciences, Lahore. All animals were kept in ventilated cages (23°C temperature, 55% humidity and light/dark cycle 12 h) with free access to water *ad libitum*. The animals were acclimatized for 1 week. The studies were performed according to methods approved by The Ethical Review Committee (ERC) at GCUF (Ref. No. GCUF/ERC/50).

### Plant collection and extraction

In March 2019, the plant material was collected from Gilgit-Baltistan, Pakistan. The plant was identified and authenticated by a botanist, Prof. Dr. Mansoor Hameed, Department of Botany, University of Agriculture, Faisalabad, Pakistan. The specimen sample was submitted at Herbarium, University of Agriculture with voucher number (31-21-01) for future reference. The plant specie has also been validated using online resources like https://powo.science.kew.org/taxon/urn:lsid:ipni.org:names:106475-1 http://www.theplantlist.org/tpl1.1/record/kew-2673508; https://www.ipni.org/n/106475-1 https://sites.google.com/site/efloraofindia/system/app/pages/search?scope=search-site&q=berberis+brandisiana; http://www.efloras.org/florataxon.aspx?flora_id=5&taxon_id=242420734). To prepare the crude extract, 1.5 kg powdered plant material was soaked in methanol and distilled water (70:30, v/v) for 7 days with occasional shaking. The maceration process was repeated thrice to obtain sufficient extract. The first filtrate was obtained by using muslin cloth and Whatman filter paper No. 1. The procedure was carried out three times. A rotary evaporator (Model: RE300 Stuart^®^ United Kingdom) was used to evaporate filtrates and to obtain extract. The percentage yield of AMEBB was obtained 13% wt/wt.

## Quantitative analysis

### Estimation of total phenolic content (TPC)

To determine the total phenolic content (TPC) of AMEBB, Folin Ciocalteu spectrophotometric method was used (Kiziltas et al., 2022b; Topal and Gulcin, 2022). Around 40 µL AMEBB and gallic acid (standard) were mixed with 1.8 mL of Folin–Ciocalteu reagent and allowed to stand at room temperature for 5 min and then sodium bicarbonate (1.2 mL, 7.5%) was added to the mixture. The mixture was allowed to stand for 60 min at room temperature and absorbance was measured at 765 nm. For each sample, measurements were carried out triplicate. Gallic acid was used as a standard. TPC were calculated as mg of gallic acid in milligram equivalent (GAE)/g of dry extract.

### Estimation of total flavonoid content (TFC)

TFC of AMEBB were estimated by using calorimetric assay. A 4 mL of distilled water was added to 1 mL of AMEBB. Then, 0.3 mL of 5% sodium nitrite (NaNo_2_) solution was added, followed by 0.3 mL of 10% aluminum chloride (AlCl_3_) solution in test tubes. Test tubes were incubated for 5 min followed by addition of 2 mL of 1 M sodium hydroxide (NaOH) in the mixture. The volume of reaction mixture was made up to 10 mL with distilled water. The mixture was thoroughly vortexed. The absorbance was measured at 510 nm. A calibration curve was prepared with catechin and the results were expressed as mg catechin equivalent (CEQ)/100 g sample (Bilgari et al., 2008).

### DPPH (1, 1-diphenyl-2picryl-hydrazyl) radical scavenging assay of AMEBB

The DPPH has been widely used for the measurement of free radical scavenging activity of samples (Durmaz et al., 2022; Gülçin et al., 2022). To prepare stock solution, 4 mg of DPPH was mixed in 100 mL of methanol. Around 2800 µL of DPPH solution was mixed with various concentrations of AMEBB. A 3 mL aliquot was filled with 200 mL of AMEBB concentrations (200, 100, 50, 25, 12.5, and 6.25 μg/mL) and DPPH. The mixture was carefully shaken before being stored at room temperature for 60 min. The OD (optical density) was measured at 517 nm using a UV spectrophotometer (Hitachi, Japan). For negative control, 2800 µL DPPH and 200 mL of methanol were used. On the other hand, methanol was used as a control. Following equation was used to calculate % age inhibition or scavenging effect:

$$\%\;age\;inhibition\;or\;scavenging\;effect=\left[\left(AC-AS\right)\;/\;AC\right]\times 100$$

Where AC denotes absorbance of negative control and AS denotes absorbance of test samples. IC_50_ values were calculated using Graph pad prism (8.4.3).

### HPLC analysis of AMEBB for detection of alkaloids

The aqueous methanolic extract of *B. brandisiana* (AMEBB) was evaluated using HPLC fingerprinting and content determination method. HPLC (Perkin Elmer, United States) was attached with Flexer Binary LC pump and UV/VS LC detector (Shelton City, 06484 United States). HPLC column (C _18_) with dimensions of 260 × 4.6 mm and a thickness of 5 µm was used. Temperature of column was 36°C. A 10 µL was injected volume of the sample. The mobile phase was methanol-water (water containing 4% acetic acid) and methanol: water ratio was 66:34 v/v. The flow rate was 1 mL/min and detectors were used at the wavelength of 290 and 250 nm. Whereas, berbamine standardization was carried out with methanol (60%) and water (40%); v/v. Quantification of berbamine in AMEBB was performed by standard method using berbamine ≥98% HPLC (Batch #. 066AZF, Glenthem Life Sciences, United Kingdom) as standard. The stock solution of plant sample was prepared by mixing 50 mg of the dry extract in a 1000 mL solution of methanol/water (70:30, v/v). The HPLC chromatogram was obtained using same mobile phase and detection wavelength as used for berbamine (Dar et al., 2014). For data analysis, software version 4.2.6410 was used.

### HPLC analysis of AMEBB for detection of Flavonoids and Phenolics

AMEBB sample was prepared for high performance liquid chromatography analysis by mixing 50 mg sample in 24 mL of methanol, 16 mL of distilled water and 10 mL of 6 M HCl. The mixture was incubated at 95°C for 2 h. Solution was filtered through membrane filter (0.45 μm nylon). Gradient HPLC (Shimadzu, Japan; SPD 10AV) was used for separation of phenolics and flavonoids from AMEBB using C_18_ (shim-pack CLC-ODS), 5 μm column (25 cm × 4.6 mm) linked with UV- visible spectrophotometer detector at wavelength of 280 nm and injector for sampling. Separation was carried out on gradient mobile phase (A: Water and acetic acid, B: Acetonitrile). Flow rate was 1 mL/min. The gradient used for solvent B was 15% for 0–15 min, 45% for 15–30 min and 100% for 35–45 min compounds were interpreted by comparing the retention time (Rt) and the UV visible peaks previously obtained by standards. External standardization was used for quantification (Shaukat et al., 2022).

### Design of experiments

Wister rats were divided into nine groups (n = 6, each) prior to dietary manipulation. For 4 weeks, six groups of animals were fed high fat diet composed of 5 kg refined wheat flour, 5 kg wheat bran, 2.25 kg fish meal, 75 g table salt, 33 g multivitamin, 150 g black treacle, 2 kg powdered milk, 500 g vegetable oil, 15 g potassium metabisulphate and 2% cholesterol/15 kg feed (Aziz et al., 2013). Following that, freshly prepared intraperitoneal injection of streptozotocin (40 mg/kg) was administered after dissolving in citrate buffer (0.1 M and pH 4.5). After 1 week, fasting blood glucose levels were measured and rats with glucose levels more than 250 mg/dL were classified as diabetic. Diabetic animals were treated as follows for 56 days. Normal control rats were fed standard diet in group I. Diabetic rats were fed high fat diet in group II. Diabetic rats were given metformin (200 mg/kg) in group III as positive control. In group IV and V: diabetic rats were treated with Berb (80 and 160 mg/kg), respectively. Group VI and VII: diabetic + AMEBB (150 and 300 mg/kg) respectively. Normal animals were given Berb (80 and 160 mg/kg) and AMEBB (160 and 300 mg/kg) in Group VIII and IX, respectively. The doses of *B. brandisiana* extract (150 and 300 mg/kg) were finalized on the basis of effective doses of similar species of same genera used in animal models (Singh and Kakkar, 2009; Pareek and Suthar, 2010; Rahimi-Madiseh et al., 2017) and, by translation of human administered dose to animal dose. In traditional system of medicine, *B. brandisiana* has been used as decoction (one teaspoon per cup) (Khan and Khatoon, 2007; Jan et al., 2008; Khan et al., 2016). One teaspoon thrice a day which is equivalent to 15g/day or 250 mg/kg. The % yield of *B. brandisiana* was found 13%, as per material to yield conversion, it became1.95 g/day/kg. For translation from human to animal dose, human dose factor 7 has been multiplied with human dose for its conversion to its respective animal dose (Nair and Jacob, 2016). It resulted as 195 mg/kg. Based on aforementioned references and calculations, we have selected lower dose as 150 mg/kg and higher dose as 300 mg/kg of *B. brandisiana*. Similarly, the doses (80 and 160 mg/kg) of berbamine were chosen from the results of our preliminary pilot experiment, performed on small number of animals (data not shown) and the results of previous animal studies (Sankaranarayanan et al., 2018; Sharma et al., 2021; Yin et al., 2022) where berbamine has been used in range of 50–200 mg/kg in different studies.

### Acute toxicity research

In accordance with OECD 425 guidelines, doses of 1000 and 2000 mg/kg were used for assessment of acute toxicity of AMEBB. After 24 h, rats were observed for toxicity indicators like agitation, lacrimation, general behavior and respiration as well as mortality (Shaukat et al., 2022).

### Estimation of body weight, food intake and fluid intake

During the experimental period, an electronic weighing balance (Sartorius-Power™, United States) was used to measure the increase in body weight of animals in each group to determine the effect of HFD feed intake. To assess the impact of diet, food intake of each rat in all groups was calculated daily. Water was provided in graduated drinking bottles and daily consumption was recorded (Sholikhah and Ridwan, 2021).

### Biochemical analysis

At 12^th^ week of experiment, animals were starved for 18 h before being euthanized followed by achievement of deep anesthesia with isoflurane (5%–10% v/wt) through inhalation in a closed chamber. For the assessment of glucose levels in blood and glycosylated hemoglobin (HbA1c) levels, blood samples were drawn through cardiac puncture in EDTA tubes (EDTA, sodium citrate, heparin). Tissues were dissected, washed in ice-cold normal saline and homogenized with phosphate buffer (0.1 M, pH 7.5). The supernatant was collected after centrifugation at 3000 /rpm for 10 min. The blood was centrifuged at 4000 /rpm for 10 min to obtain serum. The serum and tissue homogenates were stored at −80°C. Serum and homogenates were preserved for further biochemical analysis (Javaid et al., 2021).

### Estimation of glucose, insulin and glycosylated hemoglobin

Blood glucose levels were measured using a digital glucometer (EVOCHECK GM700S), while serum insulin levels were measured by ELISA kit (E-EL-R3034) sourced from Elabscience, USA, with sensitivity range of 3.75 pg/mL, and a detection range of 6.25–400 pg/mL. The reaction was observed at 450 nm wavelength. HbA1c levels were determined using a commercial kit (A1C EZ 2.0 ™) sourced from Wuxi Bio Hermes Biomedical Technology Co., Ltd. Beijing, China (Sankaranarayanan et al., 2018).

### Determination of inflammatory biomarkers

Pro-inflammatory cytokines (TNF-α, IL-6) levels were measured with ELISA kits (E-EL-H0109) and (E-EL-H0102) sourced from Elabscience, United States, with sensitivity and detection ranges are of 4.69 pg/mL and 7.81–500 pg/mL, respectively. The reaction was observed at 450 ± 2 nm wavelength. The mixture reaction was provided with 100 ul of serum in already coated wells in the ELISA plate reader (DIA source, Germany). Following the recommendations of manufacturer, TNF-α and IL-6 concentrations were calculated using the standard markers included in the assay kits. The results are presented in ng/mL (Shaukat et al., 2022).

### Estimation of adipocytokines levels in serum

The obesity biomarkers in serum, adiponectin (E-EL-H 6122), leptin (E-EL-R 0582), Elabscience, United States, and chemerin (E0864Ra), BT LAB, Birmingham, United Kingdom, levels were estimated using ELISA kits according to the instructions of manufacturer with sensitivity ranges of 0.1 ng/mL and 0.52 ng/mL, respectively. The ELISA plates were pre-coated with antibodies specific to rat LEP, ADP and CHEM maintained at 37°C. The reaction was observed at 450 ± 10 nm wavelength. Serum leptin, adiponectin and chemerin levels were measured in ng/mL (Shaukat et al., 2022).

### Determination of lipid profile, LFTs and RFTs

Lipid profile including triglycerides (TGs), total cholesterol (TC) and high-density lipoprotein (HDL) levels were measured in serum samples (Rabbi et al., 2021). To carry out standardized enzymatic procedures, commercial kits (CAT # ETI10150100-4, CAT # ETI11630100-3) sourced from Humen, Germany were used. The fraction of low-density lipoprotein (LDL) was obtained through subtracting high-density lipoprotein from total cholesterol. LFTs (ALT, AST) and RFTs (creatinine and urea) levels were measured in isolated samples of serum. The results were shown in U/L and mg/dL.

### Antioxidant studies on tissue homogenates

The antioxidant activities of tissue homogenates were determined by assessing the levels of catalase (CAT), superoxide dismutase (SOD) and melondialdehyde (MDA). All animals were euthanized followed by achievement of deep anesthesia with isoflurane (5%–10% v/wt) through inhalation in a closed chamber. The pancreas, kidney, liver, aorta and heart were removed and kept at −80°C after being washed with ice-cold normal saline. The experiments were carried out according to pre-established procedures (Javaid et al., 2021).

### Histopathological analysis

Histopathological analysis was performed using formalin (10%) to preserve the liver, pancreas, kidney, aorta and heart of rats. Organs were stained with hematoxylin and eosin followed by harvesting with a microtome (Leica, Germany) and observed under light microscope (ACCU-SCOPE 3001- LED Digital Microscope, USA) (Madić et al., 2021).

### Quantitative reverse transcription polymerase chain reaction (q RT-PCR)

q RT-PCR was used to estimate the mRNA expression of IRS-1, GLUT-4, SIRT 1, ADAM 17 and β-actin. Trizol reagent (Tri Quick Reagent, Cat #R1100) was used to homogenize frozen liver tissues and to extract total RNA. Reverse transcriptase kit of cDNA was used to acquire cDNA from approximately 2 µg of total RNA/sample (Molecular Biology, Thermoscientific, Lithuania). Amplification of cDNA was carried out using SyberGreen PCR (Molecular Biology, Thermoscientific, Lithuania). The primer stock solutions were used in accordance with the protocol of manufacturer (Macrogen, Korea). With a total volume of 20 μL, PCR for IRS-1, GLUT-4, SIRT 1, ADAM 17 and β-actin was performed in the presence of 2.5 μL of cDNA template, Sybergreen (10 μL), RNAase free water (4.5 μL), forward primer (1.5 μL) and reverse primer (1.5 μL) into each sets of the primers (The primer sequence is listed in Table 1). The annealing temperatures used for all primers were (58°C–60°C for 60 s). The 2^−ΔΔCt^ method was used to calculate the relative mRNA levels of candidate genes, which were normalized to β-actin levels and compared to untreated control group (Aljohani et al., 2022).

**TABLE 1:** List of Primers used in q RT- PCR.

Genes	Forward/reverse	Sequences	Gene accession no.
**IRS-1**	Forward	5′CCA​AGG​GCT​TAG​GTC​AGA​CA 3′	2011202-005_E7
	Reverse	5′CCA​CTT​GCA​TCC​AGA​ACT​CG 3′	
**GLUT-4**	Forward	5′ TTG​CCC​TTC​TGT​CCT​GAG​AG 3′	2011202-005_D9
	Reverse	5′CGC​TCT​CTT​TCC​AAC​TTC​CG 3′	
**ADAM17**	Forward	5′AAG​ACC​CCA​GCA​CAG​ATT​CA 3′	2011202-005_D11
	Reverse	5′GGC​TCC​CAC​TAA​CAC​TCT​GT 3′	
**SIRT 1**	Forward	5′GCA​GTA​ACA​GTG​ACA​GTG​GC 3′	2011202-005_E4
	Reverse	5′AAC​TGC​CTC​TTG​ATC​CCC​TC 3′	
***β*-actin**	Forward	5′CAC​CAT​GTA​CCC​AGG​CAT​TG 3′	2011202-005_E10
	Reverse	5′ACA​GTC​CGC​CTA​GAA​GCA​TT 3′	

Bold values are represent as IRS-1: Insulin receptor substrate- 1, GLUT-4: Glucose transporter-4, SIRT1: Sirtuin-1, ADAM 17: A disintegrin and A metalloproteinase 17, β-actin: ACTB gene.

## Results

### Total flavonoid content (TFC) and total phenolic content (TPC)

Total flavonoid and phenolic equivalent contents of AMEBB were calculated using catechin and gallic acid standard regression lines, respectively. TFC and TPC contents were found to be 77.76 mg CEQ/g dry extract weight and 89.69 mg GAE/g dry extract, respectively.

### Antioxidant activities of AMEBB

**FIGURE 1:**
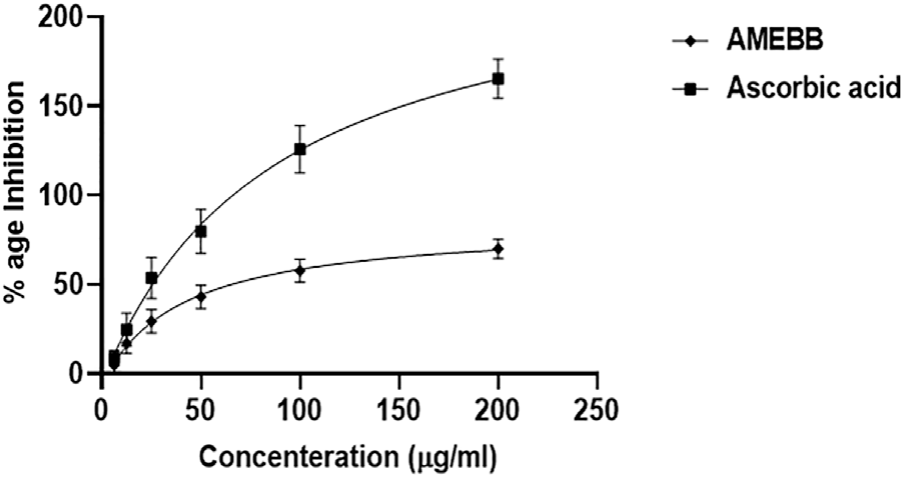
Aqueous methanolic extract *Berberis brandisiana* (AMEBB) has antioxidant DPPH radical scavenging activity *in vitro*. The values are depicted as Mean ± S.E.M. (n = 3).

AMEBB inhibited DPPH in a concentration-dependent manner with maximum of 78.15% scavenging activity at 200 µg/ml. The IC_50_ value of AMEBB was 40.32 μg/mL with 95% CI of 20.46–80.38, n = 3, similar to the IC_50_ value of ascorbic acid which was 87.09 μg/mL (51.72–156.7, n = 3) (Figure 1).

### HPLC analysis of AMEBB for detection of alkaloids

**FIGURE 2:**
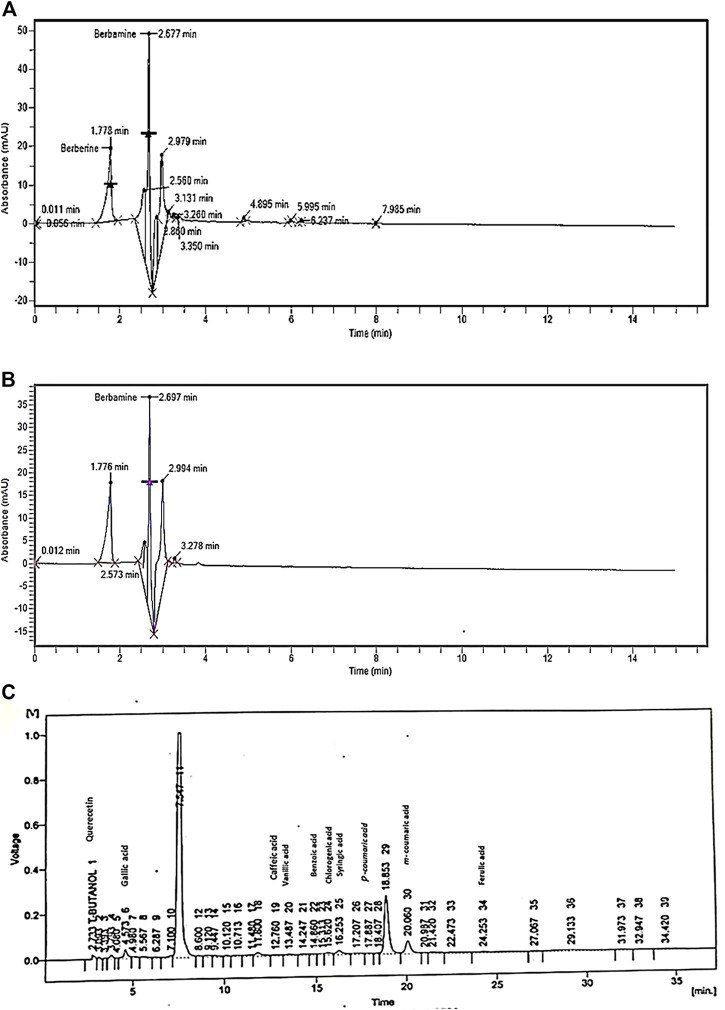
**(A)** HPLC chromatogram of aqueous methanolic extract of *Berberis brandisiana* (AMEBB) for detection of Alkaloids **(B)** HPLC fingerprints of standard (Berbamine). **(C)** HPLC chromatogram of aqueous methanolic extract of *Berberis brandisiana* (AMEBB) for detection of Flavonoids and Phenolics.

The HPLC chromatogram of the aqueous methanolic extract of *B. brandisiana* (AMEBB) displayed different metabolites with respective concentrations like, berbamine (74.95 mg) and berberine (52.15 mg) [Figures 2A, B].

### HPLC analysis of AMEBB for detection of Flavonoids and Phenolics

The HPLC chromatogram of the aqueous methanolic extract of *B. brandisiana* (AMEBB) revealed various metabolites with different concentrations in ppm like quercetin (11.14), gallic acid (23.68), caffeic acid (8.53), vanillic acid (14.43), benzoic acid (15.49), chlorogenic acid (19.89), syringic acid (13.41), *p*-coumaric acid (1.9 m), *m*-coumaric acid (15.54) and ferulic acid (25.96) [Figure 2C].

### Acute toxicity study

At highest administered dose of 2000 mg/kg of AMEBB, animals showed no signs of toxicity. All the animals were survived with no disability or deaths.

### Effect of AMEBB and Berb on body weight, food and fluid intake

**FIGURE 3:**
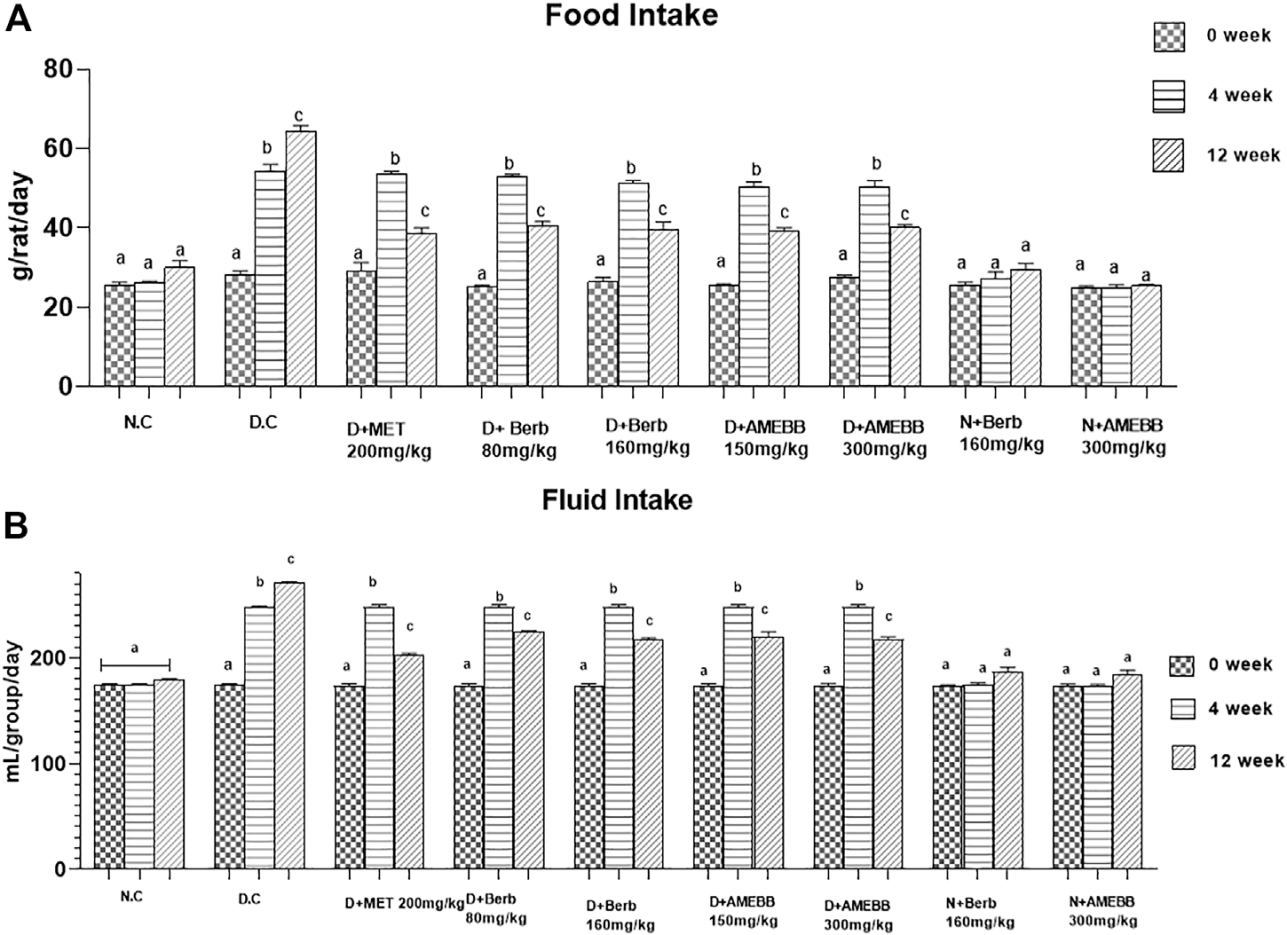
Effect of administration of *Berberis brandisiana* and berbamine on and food intake **(A)** fluid intake **(B)** in high fat diet and streptozotocin fed diabetic rats. Mean ± S.E.M (n = 6); are used to express the bars. Where N.C- Normal control, D.C- disease control, D + AMEBB–diabetic rats treated with aqueous methanolic extract of *Berberis brandisiana* (150 and 300 mg/kg), D + Berb–diabetic rats treated with aqueous solution of berbamine (80 and 160 mg/kg), D + MET-diabetic rats treated with metformin (200 mg/kg). ^a, b, c^ *p* < 0.001 shows comparisons of normal vs diabetic animals (student t-test) treatment vs diseased animals (Two Way ANOVA followed by Dunnett’s test).

Body weight of rats in groups 2–7 significantly (*p* < 0.001) increased after 28 days of high fat diet consumption. Only high fat diet and streptozotocin administered rats for 56 days, showed a noticeable (*p* < 0.01) attenuation in the body weights when compared with normal animals (Table 2). Similarly, diabetic rats consumed significantly (*p* < 0.001) more food and fluids throughout the experiment. Oral administration of AMEBB (150 and 300 mg/kg) and Berb (80 and 160 mg/kg) for 8 weeks significantly (*p* < 0.01) reduced food intake (grams) (39.20 ± 0.80, 40 ± 0.70), (40.60 ± 1.03, 39.60 ± 1.91) and fluid intake (mL) (220.2 ± 1.93, 217.6 ± 1.03), (224.4 ± 0.51, 216.8 ± 0.91) in diabetic animals compared to control (30 ± 1.73) and (179.2 ± 0.37), respectively. These findings were found similar to those on the part of metformin, a standard antidiabetic drug, administered group (Figure 3).

**TABLE 2:** Effect of administration of *Berberis brandisiana* and berbamine on body weight in high fat diet and streptozotocin-fed diabetic rats.

	Weight (gm)		
	0 week	4th week	12th week
Control (NPD)	155.6 ± 2.32	158.4 ± 1.81	158.2 ± 1.53
Diabetic (HFD + STZ)	156.6. ± 2.48	256 ± 2.821^+++^	140.8 ± 2.04^+++^
Diabetic + MET (200 mg/kg)	150.4 ± 12.01	172.8 ± 7.21^ab^	153.2 ± 5.81^ab^
Diabetic + Berb (80 mg/kg)	163 ± 0.84	189.4 ± 4.72^ab^	175.4 ± 2.68^ab^
Diabetic + Berb (160 mg/kg)	155.4 ± 1.57	180.4 ± 5.39^ab^	166.2 ± 4.99^ab/ns+^
Diabetic + AMEBB (150 mg/kg)	155.2 ± 1.16	185.2 ± 4.97^ab^	171 ± 4.92^ab^
Diabetic + AMEBB (300 mg/kg)	147.8 ± 6.42	178 ± 4.92^ab^	162 ± 4.56^ab/ns+^
Control + Berb (160 mg/kg)	152 ± 5.78	189.8 ± 3.17^ns^	201.2 ± 3.54^ns^
Control + AMEBB (300 mg/kg)	156 ± 11.31	188.6 ± 4.98^ns^	195.4 ± 4.82^ns^

The values are depicted as Mean ± S.E.M. (n = 6); +++*p* < 0.001 shows comparisons of normal vs. diabetic animals (student t-test); ^ab^ *p* < 0.01 shows comparison of treatment vs diseased animals (Two Way ANOVA, followed by Dunnett’s test); ns = non-significant; Where NPD: normal pallet diet; Diabetic: High fat diet/streptozotocin; MET: metformin; Berb: berbamine; AMEBB: aqueous methanolic extract of *Berberis brandisiana*; ns^*+*^ (non-significant)shows the comparison between the effect of low vs. high dose of *Berberis brandisiana* and berbamine (student t-test).

### Effect of AMEBB and berb on glucose, insulin and HbA1c levels

Serum insulin levels (5.54 ± 0.22 µU/mL) were significantly (*p* < 0.001) reduced in high fat diet and streptozotocin-fed diabetic rats, while blood glucose levels (346 ± 13.27 mg/dL) were significantly (*p* < 0.001) increased compared to normal control (87 ± 1.09) animals (Table 3). Administration of AMEBB (150 and 300 mg/kg) and Berb (80 and 160 mg/kg) for 8 weeks caused a significant (*p* < 0.01) improvement in serum insulin levels in µU/mL (12.11 ± 0.14, 13.08 ± 0.24) (8.3 ± 0.27, 12.92 ± 0.09) and blood glucose levels in mg/dL (156.4 ± 1.83, 135.6 ± 2.62) (242.4 ± 2.66, 158 ± 4.57), respectively compared to HFD/STZ-induced diabetic rats. HbA1c (%) levels were found significantly (*p* < 0.001) enhanced (7.66 ± 0.18) in HFD/STZ-induced diabetic rats vs. normal rats. AMEBB (150 and 300 mg/kg) and Berb (80 and 160 mg/kg) considerably (*p* < 0.01) reduced HbA1c levels (5.22 ± 0.04, 6.22 ± 0.05), (5.24 ± 0.14, 5.26 ± 0.07), respectively. However, AMEBB and Berb caused no significant changes in normal rats. AMEBB and Berb also showed marked effect at higher dose vs. its effect at lower dose in insulin and glucose levels as depicted in Table 3.

**TABLE 3:** Effect of administration of *Berberis brandisiana* and berbamine on glucose insulin, and glycated hemoglobin levels in high fat diet and streptozotocin fed diabetic rats.

	Diabetic parameters		
Groups	Glucose (mg/dL)	Insulin (µU/mL)	HbA1c (%)
Control (NPD)	87 ± 1.09	16.6 ± 0.68	4.7 ± 0.05
Diabetic (HFD + STZ)	346 ± 13.27^+++^	5.5 ± 0.22^+++^	7.6 ± 0.18^+++^
Diabetic + MET (200 mg/kg)	116.2 ± 1.07^ab^	12.9 ± 0.39^ab^	4.9 ± 0.02^ab^
Diabetic + Berb (80 mg/kg)	242.4 ± 2.66^ab^	8.3 ± 0.27^ab^	5.3 ± 0.14^ab^
Diabetic + Berb (160 mg/kg)	158 ± 4.57^ab/***^	13 ± 0.09^ab/***^	5.3 ± 0.07^ab/ns*+*^
Diabetic + AMEBB (150 mg/kg)	156.4 ± 1.83^ab^	12.1 ± 0.14^ab^	5.2 ± 0.04^ab^
Diabetic + AMEBB (300 mg/kg)	135.6 ± 2.62^ab/***^	13.1 ± 0.24^ab/**^	5 ± 0.05^ab/**^
Control + Berb (160 mg/kg)	86.4 ± 0.75 ^ns^	16.3 ± 0.04^ns^	5.5 ± 0.08^ns^
Control + AMEBB (300 mg/kg)	84 ± 0.78^ns^	16.2 ± 0.22^ns^	4.5 ± 0.06^ns^

The values are depicted as Mean ± S.E.M. (n = 6); +++*p* ˂ 0.001 shows comparisons of normal vs diabetic animals (student t-test); ^a^ *p* < 0.05 and; ^ab^ *p* < 0.01 show comparison of treatment vs diseased animals (Dunnett’s test followed One Way ANOVA); ns = non-significant; Where NPD: normal pallet diet; Diabetic: High fat diet/streptozotocin; MET: metformin; Berb: berbamine; AMEBB: aqueous methanolic extract of *berberis brandisiana*; HbA1c: glycosylated hemoglobin; ns^+^ (non-significant), ***p < 0.001 and ** *p <* 0.01 show the comparison between the effect of low vs. high dose of *Berberis brandisiana* and berbamine (student t-test).

### Effect of AMEBB and Berb on inflammatory biomarkers

**FIGURE 4:**
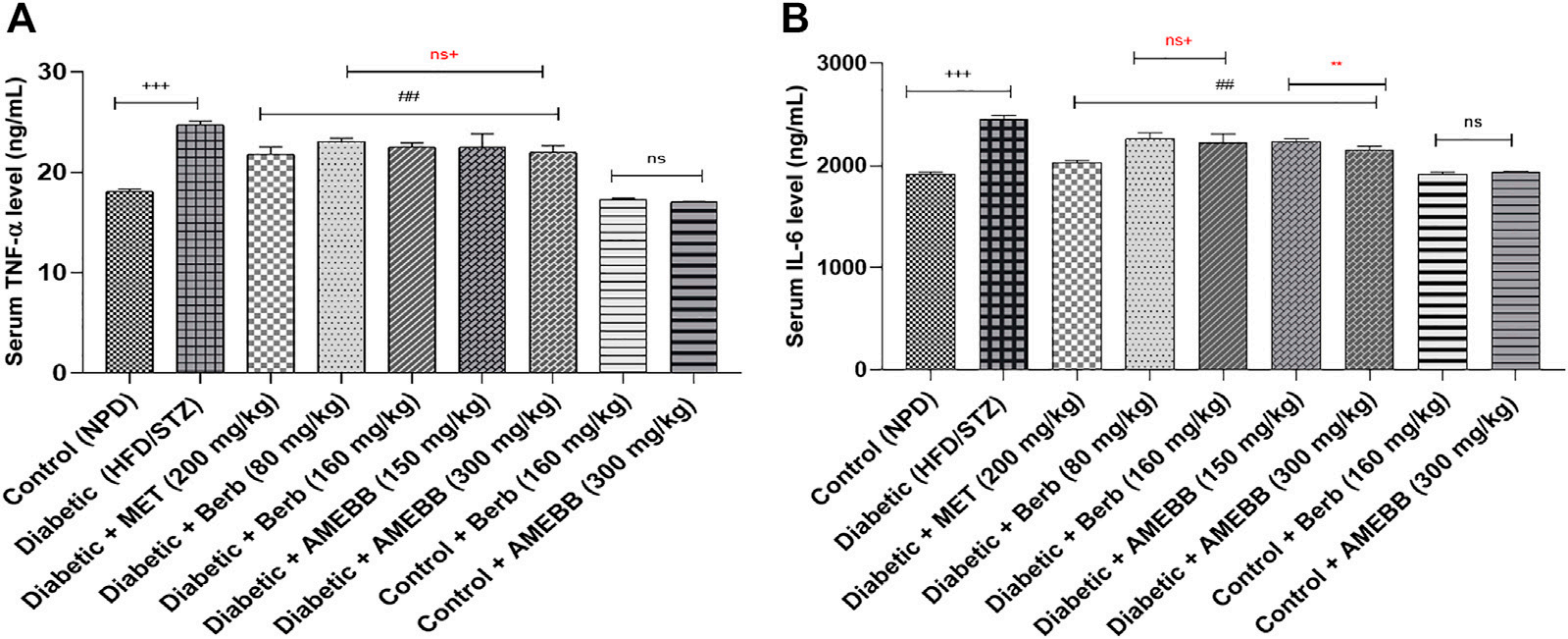
Effect of administration of *Berberis brandisiana* and berbamine on TNF- α **(A)** and IL-6 **(B)** in high fat diet and streptozotocin fed diabetic rats. The values are given as Mean ± S.E.M. (n = 6); ^+++^ *p* < 0.001 shows comparisons of normal vs diabetic animals (student t-test); ^###^ *p* < 0.001 shows comparison of treatment vs diseased animals (Dunnett’s test followed One Way ANOVA); ns = non-significant; Where NPD: normal pallet diet; Diabetic: High fat diet/streptozotocin; MET: metformin; Berb: berbamine; AMEBB: Aqueous methanolic extract of *Berberis brandisiana*; IL-6: interleukin 6: TNF-α: Tumor necrosis factor alpha. ns^+^ (non-significant) and ^**^ *p <* 0.01 *s*how the comparison between the effect of low vs. high dose of *B. brandisiana* and berbamine (student t-test).

When AMEBB (150 and 300 mg/kg) and Berb (80 and 160 mg/kg) were given to HFD/STZ-induced diabetic rats, these presented marked (*p* < 0.001) decline in serum TNF-α (20.39 ± 0.17, 19.20 ± 0.37) (20.43 ± 0.39, 19.95 ± 0.27) and IL-6 (ng/mL) levels compared to only HFD/STZ-exposed rats. Administration of AMEBB and Berb showed no marked difference in serum TNF-α and IL-6 levels of normal rats. AMEBB and Berb also showed marked effect at higher dose vs. its effect at lower dose in serum IL-6 levels as depicted in Figure 4.

### Effect of AMEBB and Berb on adipocytokines levels

**FIGURE 5:**
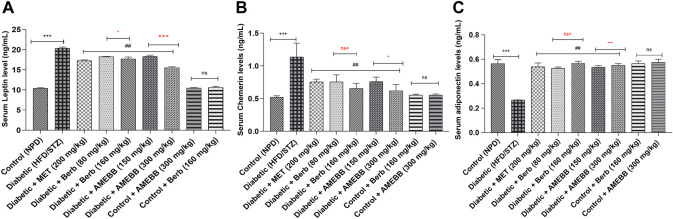
Effect of administration of *Berberis brandisiana* and berbamine on leptin **(A)**, Adiponectin **(B)** and Chemerin **(C)** in high fat diet and streptozotocin fed diabetic rats. The values are given as Mean ± S.E.M. (n = 6); ^+++^ *p* < 0.001 shows comparisons of normal vs diabetic animals (student t-test); ^##^ *p* < 0.01 shows comparison of treatment vs diseased animals (One Way ANOVA followed by Dunnett’s test); ns = non-significant; Where NPD: normal pallet diet; Diabetic: High fat diet/streptozotocin; MET: metformin; Berb: berbamine; AMEBB: Aqueous methanolic extract of *Berberis brandisiana*; ns^+^ (non-significant), ***p < 0.001 and *p <* 0.05 *s*how the comparison between the effect of low vs. high dose of *B. brandisiana* and berbamine (student t-test).

HFD/STZ-induced diabetic rats exhibited marked (*p* < 0.001) increase in serum leptin (20.38 ± 0.11) and chemerin (1.48 ± 0.15) levels compared to normal rats. In treated animals, AMEBB (150 and 300 mg/kg) and Berb (60 and 180 mg/kg) significantly (*p* < 0.01) decreased leptin (18.27 ± 0.15, 15.52 ± 0.10) (18.21 ± 0.04, 17.71 ± 0.19) and chemerin (0.76 ± 0.03, 0.62 ± 0.04), (0.80 ± 0.04, 0.60 ± 0.03) levels, respectively, compared to only HFD/STZ-exposed rats. However, a noticeable (*p* < 0.001) decrease in adiponectin (0.21 ± 0.04) levels in ng/mL were recorded in HFD/STZ-exposed rats compared to control animals. Treatment with AMEBB and Berb at both doses caused improvement in adiponectin (0.53 ± 0.01, 0.55 ± 0.01), (0.52 ± 0.01, 0.56 ± 0.01) levels respectively, similar to the effect of metformin. There was no significant modification in normal rats when treated with AMEBB and Berb. AMEBB and Berb also showed marked effect at higher dose vs. its effect at lower dose effects in serum, leptin, chemerin and adiponectin levels as displayed in Figure 5.

### Effect of AMEBB and Berb on oxidative stress

**FIGURE 6:**
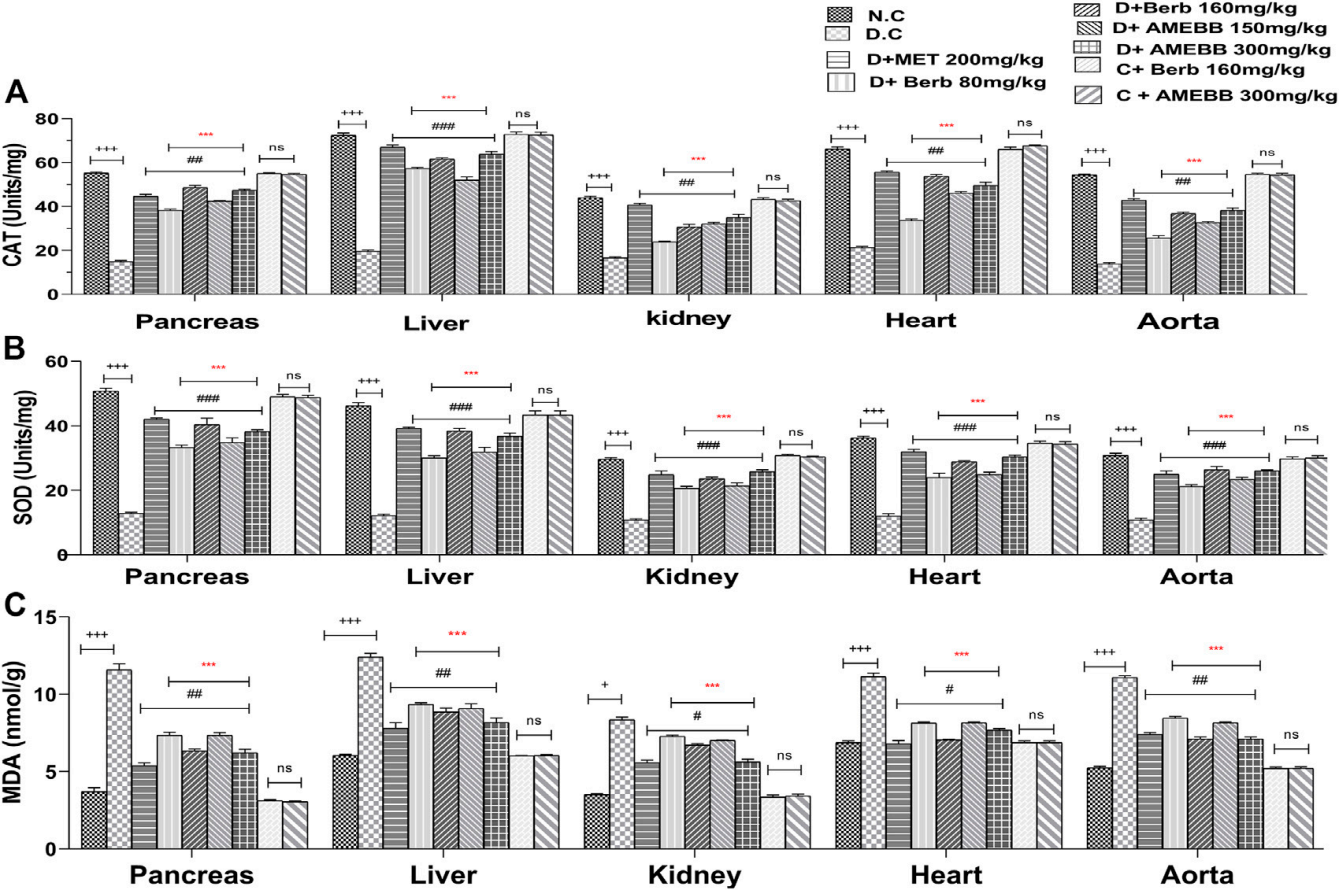
Effect of administration of *Berberis brandisiana* and berbamine on CAT **(A)**, SOD **(B)** and MDA **(C)** in high fat diet and streptozotocin-fed diabetic rats The values are given as Mean ± S.E.M. (n = 6); ^+++^ *p* ˂ 0.001 shows comparisons of normal vs diabetic animals (student t-test); ^*#*^ *p* < 0.05, ^##^ *p* ˂ 0.01 and ^###^ *p* ˂ 0.001, ns = non-significant show comparison of treatment vs diseased animals and comparison of normal vs normal treated animals (One Way ANOVA followed by Dunnett’s test); Where NPD: normal pallet diet; Diabetic: High fat diet/streptozotocin; MET: metformin; Berb: berbamine; AMEBB: Aqueous methanolic extract of *Berberis brandisiana*; Catalase (CAT), superoxide dismutase (SOD), malondialdehyde (MDA); ***p < 0.001 *s*hows the comparison between the effect of low vs. high dose of *B. brandisiana* and berbamine (student t-test).

Figure 6 depicts oxidative damage markers in liver**,** pancreas, kidney, aorta and heart tissue homogenates. Treatment of AMEBB and Berb to HFD/STZ-induced diabetic rats showed a noticeable (*p* < 0.001) increase in serum antioxidant enzymes (CAT and SOD in Units/mg) in pancreas (42.4 ± 0.24,47.4 ± 0.51), (38.2 ± 0.583, 48.6 ± 1.03) and liver (52 ± 1.41, 63.2 ± 0.51), (57.2 ± 0.58, 61.6 ± 1.24) and superoxide dismutase levels in pancreas (34.8 ± 1.46,38.2 ± 0.58), (33.2 ± 0.80, 40.4 ± 1.96) and liver (31.8 ± 1.52,36.8 ± 0.96), (30 ± 0.70, 38.4 ± 0.81), kidney, heart and aorta homogenates when compared with only HFD/STZ-exposed animals. Administration of AMEBB (150 and 300 mg/kg) and Berb (80 and 160 mg/kg) to HFD/STZ-fed diabetic rats caused significant (*p* < 0.01) decrease in MDA (nmol/g) levels in pancreas (7.34 ± 0.17, 6.22 ± 0.22), (7.34 ± 0.20, 6.34 ± 0.11) and in liver (9.08 ± 0.31, 8.18 ± 0.29), (9.34 ± 0.10, 8.86 ± 0.24) homogenates compared to the control group of animals. AMEBB and Berb also showed marked (*p* < 0.001) effect at higher dose vs. its effect at lower dose in tissue homogenates of pancreas, liver, kidney, heart and aorta (Figure 6).

### Effect of AMEBB and Berb on serum biochemical parameters

Administration of AMEBB (150 and 300 mg/kg) and Berb (80 and 160 mg/kg) in hyperglycemic rats revealed a marked (*p* < 0.001) attenuation in triglycerides (TG), total cholesterol (TC), low-density lipoprotein (LDL), aspartate aminotransferase (AST), alanine transaminase (ALT), urea and creatinine levels compared to only HFD/STZ-exposed rats, similar to the effect of metformin. When AMEBB and Berb were administered to HFD/STZ-exposed diabetic rats, it caused a marked (*p* < 0.001) increase in HDL levels compared to only HFD/STZ challenged rats (Tables 4, 5).

**TABLE 4:** Effect of administration of *Berberis brandisiana* and berbamine on Lipid Profile in high fat diet and streptozotocin-fed diabetic rats.

	Lipid profile			
Groups	TC (mg/dL)	TG (mg/dL)	HDL (mg/dL)	LDL (mg/dL)
Control (NPD)	121.6 ± 0.51	103.2 ± 0.97	46.6 ± 0.81	75 ± 0.20
Diabetic (HFD + STZ)	315.2 ± 4.49^+++^	204.2 ± 0.66^+++^	23 ± 0.58^+++^	292 ± 0.40^+++^
Diabetic + MET (200 mg/kg)	174.6 ± 1.21 ^ab^	116.6 ± 0.68 ^ab^	32.60 ± 0.40 ^ab^	142 ± 0.20 ^ab^
Diabetic + Berb (80 mg/kg)	183.6 ± 0.87 ^ab^	124.8 ± 0.37 ^ab^	35.8 ± 0.66 ^ab^	148 ± 0.37 ^ab^
Diabetic + Berb (160 mg/kg)	172.8 ± 1.53 ^ab/***^	117.4 ± 0.93 ^ab/***^	32.4 ± 0.25 ^ab/***^	140 ± 0.32 ^ab/***^
Diabetic + AMEBB (150 mg/kg)	185 ± 1.30 ^ab^	125.8 ± 0.74 ^ab^	36 ± 0.55 ^ab^	149 ± 0.25 ^ab^
Diabetic + AMEBB (300 mg/kg)	175.8 ± 1.07 ^ab/***^	115.2 ± 0.58 ^ab/***^	32.8 ± 0.66 ^ab/***^	143 ± 0.51 ^ab/***^
Control + Berb (160 mg/kg)	121 ± 0.32 ^ns^	105.2 ± 0.37 ^ns^	44.6 ± 1.44^+^	76.4 ± 1.24 ^ns^
Control + AMEBB (300 mg/kg)	121 ± 0.22 ^ns^	105.8 ± 0.37 ^ns^	45 ± 0.71^+^	76 ± 0.92 ^ns^

The values are depicted as Mean ± S.E.M. (n = 6); +++*p* < 0.001 shows comparisons of normal vs diabetic animals (student t-test); ^a^ *p* < 0.05 and ^ab^ *p* < 0.01 show comparison of treatment vs diseased animals (One Way ANOVA, followed by Dunnett’s test); ns = non-significant; Where NPD: normal pallet diet; Diabetic: High fat diet/streptozotocin; MET: metformin; Berb: berbamine; AMEBB: aqueous methanolic extract of *Berberis brandisiana*; total cholesterol (TC) triglycerides (TGs), high-density lipoprotein (HDL) low-density lipoprotein (LDL); ***p < 0.001 shows the comparison between the effect of low vs. high dose of *B. brandisiana* and berbamine (student t-test).

**TABLE 5:** Effect of administration of *Berberis brandisiana* and berbamine on LFTs and RFTs in high fat diet and streptozotocin-fed diabetic rats.

Groups	LFTs and RFTs			
	AST (U/L)	ALT (U/L)	Urea (mmol/L)	Creatinine (µmol/L)
Control (NPD)	113.8 ± 0.58	81.4 ± 0.510	12.3 ± 0.20	34.6 ± 0.25
Diabetic (HFD + STZ)	271 ± 1.11^+++^	181 ± 0.45^+++^	36.2 ± 1.24^+++^	73.8 ± 1.46^+++^
Diabetic + MET (200 mg/kg)	166.4 ± 0.82 ^ab^	104.4 ± 0.68 ^ab^	14.6 ± 0.40 ^ab^	38.8 ± 0.37 ^ab^
Diabetic + Berb (80 mg/kg)	175.4 ± 0.25 ^ab^	132.8 ± 0.37 ^ab^	17.8 ± 0.12 ^ab^	52.2 ± 0.38 ^ab^
Diabetic + Berb (160 mg/kg)	173.2 ± 0.37 ^ab/***^	120.6 ± 1.40 ^ab/***^	16.2 ± 0.12 ^ab/***^	41 ± 0.45 ^ab/***^
Diabetic + AMEBB (150 mg/kg)	177.6 ± 1.08 ^ab^	132 ± 0.55 ^ab^	18 ± 0.32 ^ab^	51.6 ± 0.51^ab^
Diabetic + AMEBB (300 mg/kg)	175.6 ± 0.51 ^ab/ns*+*^	121.8 ± 156 ^ab/***^	16.2 ± 0.242^ab/***^	42.3 ± 0.54 ^ab***^
Control + Berb (160 mg/kg)	114.2 ± 0.37 ^ns^	80.8 ± 0.37 ^ns^	12.2 ± 0.20 ^ns^	34 ± 0.55 ^ns^
Control + AMEBB (300 mg/kg)	114 ± 0.32 ^ns^	80 ± 0.37 ^ns^	12.6 ± 0.25 ^ns^	34 ± 0.55 ^ns^

The values are depicted as Mean ± S.E.M. (n = 6); +++*p* < 0.001 shows comparisons of normal vs diabetic animals (student t-test); ^ab^ *p* < 0.01 shows comparison of treatment vs. diseased animals (One Way ANOVA, followed by Dunnett’s test); ns = non-significant; Where NPD: normal pallet diet; Diabetic: High fat diet/streptozotocin; MET: metformin; Berb: berbamine; AMEBB: aqueous methanolic extract of *berberis brandisiana*; aspartate transaminase (AST) alkaline phosphatase (ALT); ns^+^ (non-significant) and ***p < 0.001 *s*how the comparison between the effect of low vs. high dose of *B. brandisiana* and berbamine (student t-test).

### Effect of AMEBB and Berb on histopathology of organs

**FIGURE 7:**
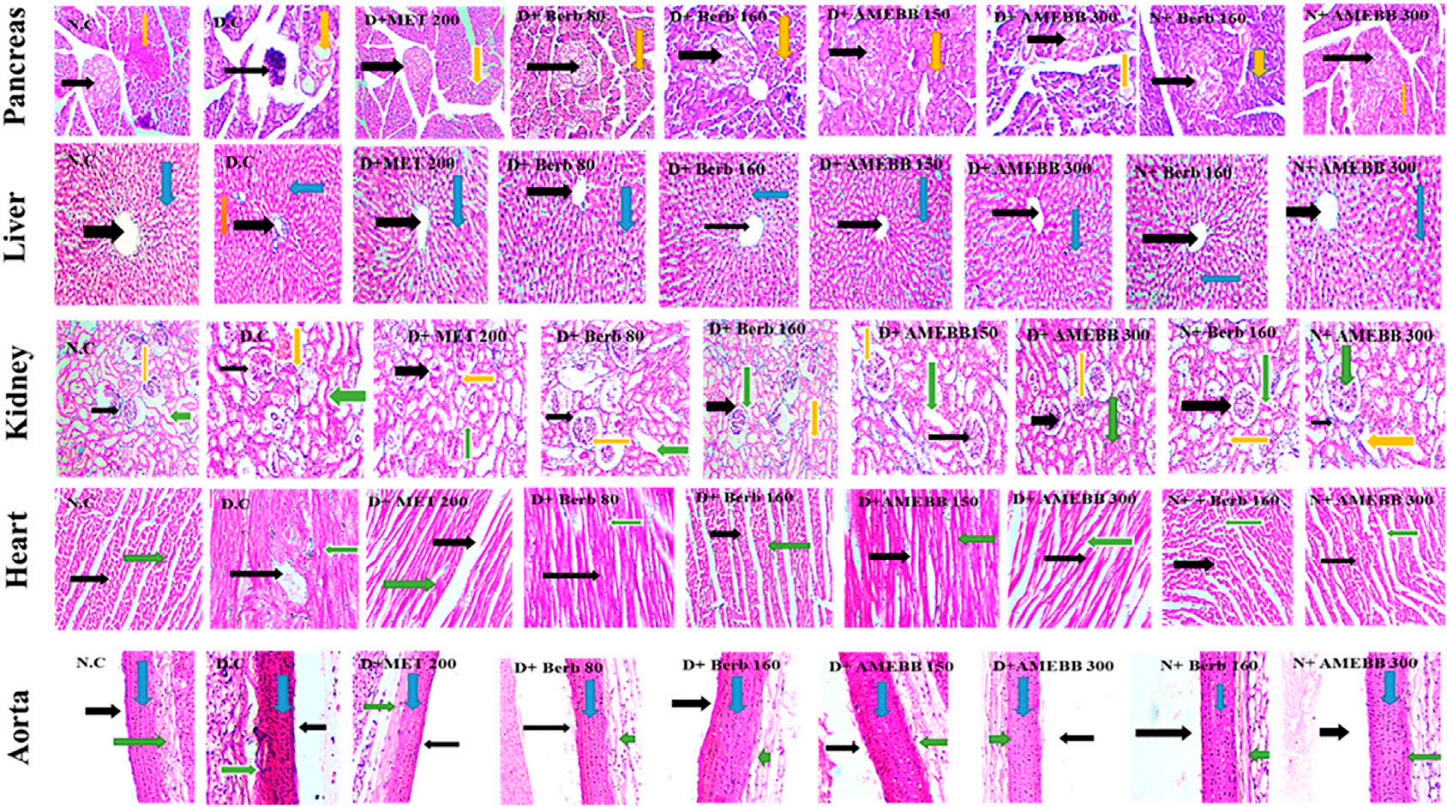
Photomicrographic representation of pancreatic tissue, Liver, kidney, heart and aorta sections showing the influence of administration of Berb: Berbamine (80 and 160 mg/kg body weight) and AMEBB: Aqueous methanolic extract of *Berberis brandisiana* (150 and 300 mg/kg body weight) respectively in HFD/STZ-induced diabetic (D.C) and normal rats (N.C). Pancreas: Black and yellow arrows respectively show pancreatic islets and exocrine parts of pancreas. Liver: Black, red and blue arrows respectively show: central vein, plates of hepatic cells and sinusoids. Kidney: Black, yellow and green arrows respectively show bowman capsule, glomerulus and distil proximal convoluted tubule. Heart: Black, and green arrows respectively show nuclei of cardiomyocytes and myofibers. Aorta: Black, blue and green arrows respectively show lumen, tunica intima, and tunica adventia.

The histopathological analysis of pancreas, liver, kidney, heart, and aortic sections from normal control and HFD/STZ-induced diabetic rats are presented in Figure 7.

Administration of AMEBB and Berb displayed an improvement in texture of islets of Langerhans in pancreas, betterment in disarray of hepatocytes, accumulation of fat droplets and inflammation of sinusoids in lives tissues. Restoration of normal glomerulus and absence of inflammatory cells was observed in kidney tissues. Isolated heart tissue of treated rats showed effective restoration of myocytes and attenuation of inflammation. Whereas, aortic tissue sections of treated animals, showed significant upgrading in the deposition of elastic fibers and reduction in lipid deposition in tunica intima as detailed in Figure 7.

### Effect on gene expression

**FIGURE 8:**
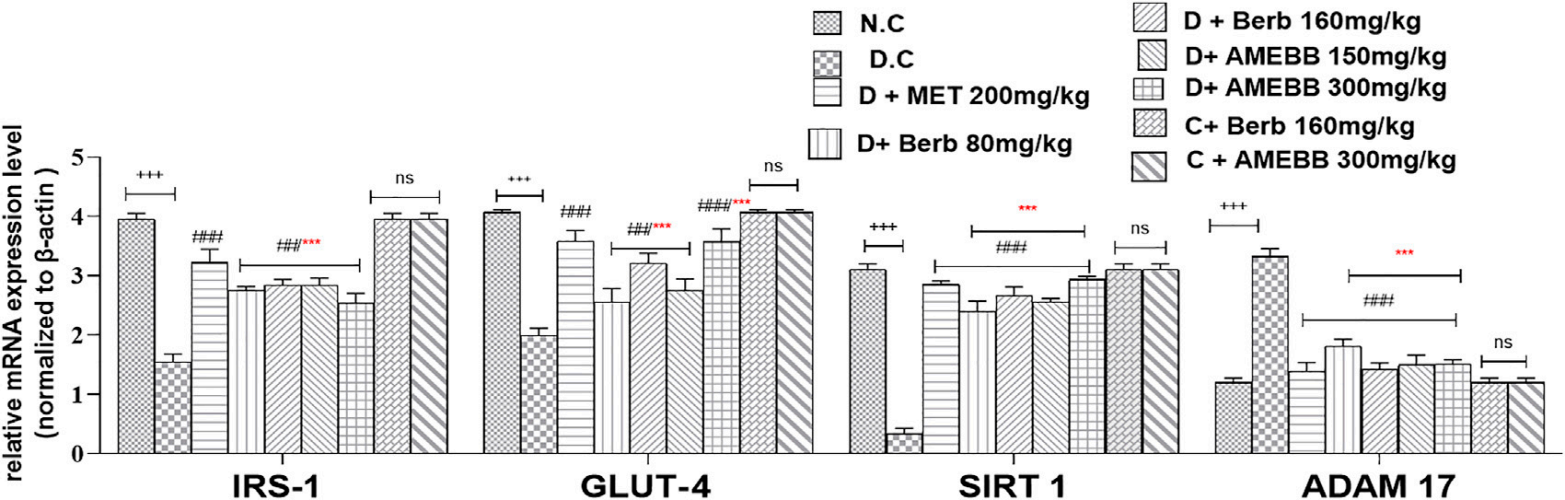
Effect of administration of *Berberis brandisiana* and berbamine on IRS-1, GLUT-4, SIRT 1, ADAM 17 mRNA level in high fat diet and streptozotocin fed diabetic rats. Values are expressed as Mean ± S.E.M, n = 6; ^+++^ *p* < 0.001 shows comparisons of normal vs diabetic animals (student t-test). ^##^ *p* < 0.01 and ^###^ *p* < 0.001 show comparison of treatment vs diseased animals (One Way ANOVA followed by Dunnett’s test); ns = non-significant; Where NPD: normal pallet diet; Diabetic: High fat diet/streptozotocin; MET: metformin; Berb: berbamine; AMEBB: Aqueous methanolic extract of *Berberis brandisiana;* IRS-1: insulin receptor substrate-1; GLUT-4: glucose transporter −4; SIRT 1; sirtuin 1; ADAM 17: A disintegrin and A metalloproteinase 17; ***p < 0.001 *s*hows the comparison between the effect of low vs. high dose of *B. brandisiana* and berbamine (student t-test).

The mRNA expressions of IRS-1, GLUT-4, SIRT 1, and ADAM 17 were assessed using Real time qPCR. The mRNA levels of ADAM 17 gene were markedly (*p* < 0.001) upregulated by 3.33 fold compared to data of control animals. When compared to the normal control group, IRS-1, GLUT-4, and SIRT 1 were significantly (*p* < 0.001) downregulated by 1.5, 1.9, and 0.33 fold, respectively, in HFD/STZ-exposed groups. In comparison to HFD/STZ-induced diabetic groups, administration of AMEBB and Berb upregulated IRS-1, GLUT-4, and SIRT 1, while caused downregulation of ADAM 17 in liver tissues, results in restoration of the expression of genes of interest towards normal. AMEBB and Berb also showed marked effect at higher dose vs. its effect at lower dose in mRNA levels of IRS-1, GLUT-4 and SIRT1 (Figure 8).

### Statistical analysis

Graph Pad Prism 8.4.3 was used for analysis and graphical presentation of the data. Values were displayed as mean ± standard error of the mean (SEM). For comparison of the data in different groups One-way analysis of variance (ANOVA) followed by Dunnett’s test or Two-way analysis of variance (ANOVA) followed by Dunnett’s test for multiple comparisons were used. *p* < 0.05 was considered significantly different.

## Discussion

Diabetes related morbidity and mortality are posing a continuous harmful impact on health systems around the globe. Additionally, nutritional fat consumption reduces glucose utilization mediated by insulin and endorses insulin resistance in diabetics. Further, 40 mg/kg streptozotocin administration to HFD-fed rats cause β-cells necrosis, resulting in deficiency of insulin and development of diabetes (Pratiwi et al., 2021). To assess the efficacy of a test material for its insulin sensitizing and/or insulin secretory properties, high fat and streptozotocin-fed animal model is considered appropriate. Similar models have been used in numerous labs (Sakashita et al., 2021). Traditional use of *B. brandisiana* in the treatment of diabetes (Jan et al., 2008) provide basis for further studies to strengthen its potential use as antidiabetic agent. *B. brandisiana* has also been found enriched with berbamine (Khan et al., 2016). *B. brandisiana* and berbamine are known for their antioxidant, anti-inflammatory, hepatoprotective and cardiovascular beneficial effects (Sankaranarayanan et al., 2018). This study has been designed to assess the ameliorating potential of the aqueous methanolic extract of *B. brandisiana* (AMEBB) and berbamine in HFD/STZ-induced diabetic rats.

The HPLC analysis showed variety of phenolic acids and flavonoids in AMEBB including, quercetin, gallic acid, caffeic acid, benzoic acid, ferulic acid, chologenic acid, syringic acid, m-coumaric acid, p-coumaric acid and ferulic acid. Most of these metabolites (flavonoids, m-coumaric acid, p-coumaric acid, quercetin, gallic acid) are known to boost insulin sensitivity, slow down the rate, digestion and absorption of sugar, hence supporting their protective effects in diabetes (Mihaylova et al., 2018; Sankaranayanan et al., 2018).

Administration of high fat diet for 56 days along with streptozotocin develop glucose intolerance, insulin resistance, β-cell destruction, alterations in inflammatory biomarkers (TNF-α, IL-6), adipocytokines (leptin, chemerin and adiponectin) levels, oxidative stress biomarkers (CAT,SOD and MDA) and diabetic candidate genes like IRS-1. GLUT-4, SIRT 1 and ADAM 17. Treatment with AMEBB and Berb to HFD/STZ-administered rats from 28^th^ day of induction period, resulted in weight gain, increased food consumption and fluid intake compared to non-diabetic rats during the experimental period. Although the food intake of diabetic rats was increased during the experimental period, the weight gain was significantly reduced. It has been reported that diabetes is associated with weight loss, polydipsia, polyphagia and polyuria (Peng et al., 2021). Energy metabolism is compromised in hepatic tissue, thus a low energy state is stimulated for satiety center and food consumption is increased in diabetes mellitus. As hepatic energy influences feeding behavior, hence in turn affects body weight (Rawlinson and Andrews, 2021). High fat diet-fed rats showed increase in body weight over a 4-week period due to its deposition in a variety of body fat packs. Weight gain was significantly (*p* < 0.01) reduced during the experimental period while food intake of diabetic rats was increased. In diabetic rats, the inability to use carbohydrates as an energy source, combined with poor glycemic control, causes extreme protein catabolism in order to supply amino acids for gluconeogenesis, resulting in muscle deterioration and weight loss during insulin deficiency (Kumar et al., 2021). Flavonoids have previously been identified as active metabolites of AMEBB, and are known for their slimming properties (Yin et al., 2022). In comparison to normal control, osmotic diuresis increases fluid consumption only in high fat diet (HFD) and streptozotocin (STZ) exposed rats. Oral administration of AMEBB and Berb considerably (*p* < 0.01) improved body weight and normalized food and fluid consumption in treated rats, indicating an improvement in glycemic control as previously reported (Sankaranarayanan et al., 2018).

Persistent hyperglycemia causes non-enzymatic glycation of proteins, including lens crystalline protein and hemoglobin. In uncontrolled diabetes, glycosylation of hemoglobin occurs gradually and is proportional to fasting blood glucose levels. According to Babaya et al. (2021), a persistent increase in HbA1c level was related with failure of β-cell function. Under hyperglycemic condition due to HFD/STZ-exposure, levels of HbA1c were higher in diabetic rats vs. normal control rats. When compared to only HFD/STZ-exposed rats, Berb and AMEBB administration decreased glucose and HbA1c levels in diabetic rats. Earlier reports have revealed that antioxidant constituents are known to inhibit glycation of protein associated with diabetes (Sarmah and Roy, 2021). These aspects offer support to the antidiabetic effect of AMEBB and Berb. HPLC analysis confirmed the presence of flavonoids such as gallic acid, quercetin and polyphenols as active plant metabolites. The presence of such advantageous metabolites contributes to the assessed benefits of AMEBB as well.

Present study showed administration of AMEBB and Berb significantly (*p* < 0.01) decreased serum TNF-α and IL-6 levels compared to only HFD/STZ-administered rats. Administration of HFD for 4 weeks helps in progression of obesity (Leite et al., 2021). Obese adipose tissues secrete a number of pro-inflammatory cytokines, including TNF-α and IL-6. There is emerging evidence that increased pro-inflammatory cytokine release is linked to development of insulin resistance because of β-cell degradation and has been reported to increase diabetes related complication (Heo et al., 2021). The AMEBB and Berb significantly modified TNF-α and IL-6 levels which is also supported by earlier studies on the part of other botanical drugs (Zheng et al., 2021).

Adipocytokines are secreted by adipose tissues and are known to contribute in defective insulin secretion and action, resulting in peripheral insulin resistance (Deng et al., 2021). Obesity-related diseases have been linked to the deficiency of adiponectin, such as diabetes, insulin resistance and cardiovascular diseases. Findings of our study are in line with earlier study of Achari and Jain. (2017). Leptin, also known as “anorexigenic” hormone, promotes oxidation of fatty acids and lipolysis while preventing lipogenesis. Other than obesity, hyperleptinemia has been linked to insulin resistance. Chronic hyperlipidemia imparts negative impact on leptin function as decribed by López-Jaramillo et al. (2014). Chemerin is a relatively new adipokine that has been discovered to have endocrine, paracrine, and autocrine functions. It also plays important role in lipid and glucose homeostasis, angiogenesis, inflammation, immune modulation and blood pressure regulation (Roman et al., 2012). The current study found a link between AMEBB and Berb supplementation and adipocytokines levels in diabetic rats. Lower levels of leptin and chemerin and higher level of adiponectin were observed in diabetic rats treated with AMEBB and Berb. AMEBB and Berb showed dose dependent effects.

The CAT and SOD levels were markedly (*p* < 0.01) increased in AMEBB (150 and 300 mg/kg) and Berb (80 and 160 mg/kg) treated groups compared to HFD/STZ-exposed rats. SOD and CAT are antioxidant enzymes that serve as the first line of defense against ROS in cells, scavenging the toxic intermediate of partial oxidation. Decrease in the levels of these antioxidant enzymes results in an additional molecular oxygen and hydrogen peroxide, which produces reactive hydroxyl free radicals and leads to the lipid peroxidation (Repetto et al., 2012). Superoxide dismutase enzyme protects cells from reactive oxygen species by scavenging molecular oxygen, which causes damage to membrane and biological structure (Ighodaro and Akinloye, 2018). The decrease in catalase activity happens due to the enzyme being inactivated by glycation. AMEBB and Berb treatment increased the levels of SOD and CAT in diabetic rats compared to only HFD/STZ-induced diabetic rats. Indeed, the return of SOD levels promoted by AMEBB and Berb may hasten the superoxide dismutation to hydrogen peroxide, which is rapidly detached by CAT, protecting diabetic animal tissues from highly toxic hydroxyl ion free radicals, result in avoiding lipid peroxidation. These results are also in line with earlier study of Aboonabi et al. (2014).

An abnormal blood lipid profile is another symptom of insulin resistance. The current study found that diabetic rats had higher total cholesterol (TC), triglycerides (TG), low-density lipoprotein (LDL), and lower HDL levels. These changes are endorsed to an increase in free fatty acid flux into the liver as a result of insulin deficiency or insulin resistance which results in an excess accumulation of fatty acid in liver and conversion to triglycerides (Adiels et al., 2008). The inability of insulin to inhibit the liberation of free fatty acids results in increased VLDL production in the liver (Pilz and März, 2008). When VLDL and TG levels rise, the activation of lipoprotein lipase and lecithin acyl-cholesterol transferase, results in decrease HDL and an increase in the concentration of LDL particles (Sharma et al., 2010). High levels of cholesterol in HFD/STZ-induced diabetic animals may be due to increased dietetic cholesterol captivation from the small intestine succeeding high fat diet consumption in the diabetic condition (Naidu et al., 2015). Furthermore, hypertriglyceridemia may increase triglycerides absorption in the form of chylomicrons as a result of over consumption of fat-rich diet. The AMEBB and Berb treatment showed noticeable (*p* < 0.01) decrease in, TG, TC, LDL and increase in HDL levels. Dietary polyphenols, on the other hand, effectively reduce the amount of lipoprotein rich in triglycerides and are linked to oxidative stress in postprandial and fasting conditions (Vries et al., 2014).

High concentrations of AST, ALT are typical indicators of liver dysfunction often observed in HFD-fed and STZ induced diabetic animals. Observed increase in AST and ALT levels may be due to the outflow of enzymes from cytosol of hepatocytes into the blood stream. The AMEBB and Berb treatment significantly (*p* < 0.01) decreased levels of these elevated enzymes and subsequently relieved liver injury. Increased levels of creatinine and urea in the serum of diabetic animals were found to be strongly correlated with renal damage. Renal tissue depletion in diabetic rats was caused by the production of reactive oxygen species as a result of elevated free radical concentrations in these tissues (El-Alfy et al., 2005). In our study, diabetic rats treated with AMEBB and Berb had significant (*p* < 0.01) reduction in serum urea and creatinine levels and restored renal structural parameters in diabetic rats, possibly by neutralizing free radicals in biological systems.

The histopathological data revealed that treatment with AMEBB and Berb improved in β-cell mass and islets of Langerhans in pancreatic tissues when compared with only HFD/STZ-exposed rats. Histograms of diabetic rat hepatic sections showed hepatocytes degeneration and inflamed sinusoids. Treatment with AMEBB and Berb protected hepatic lesions and inflammation. In diabetic group, kidney tissue sections showed degenerated renal tubules and inflammation, whereas AMEBB and Berb treatment resulted in revival of renal tubules damage and inflammation. Heart tissue of treated rats showed effective restoration of myocytes and attenuation of inflammation. Whereas, aortic tissues of treated groups showed significant upgrading in deposition of elastic fibers and reduction in lipid deposition in tunica intima.

**FIGURE 9:**
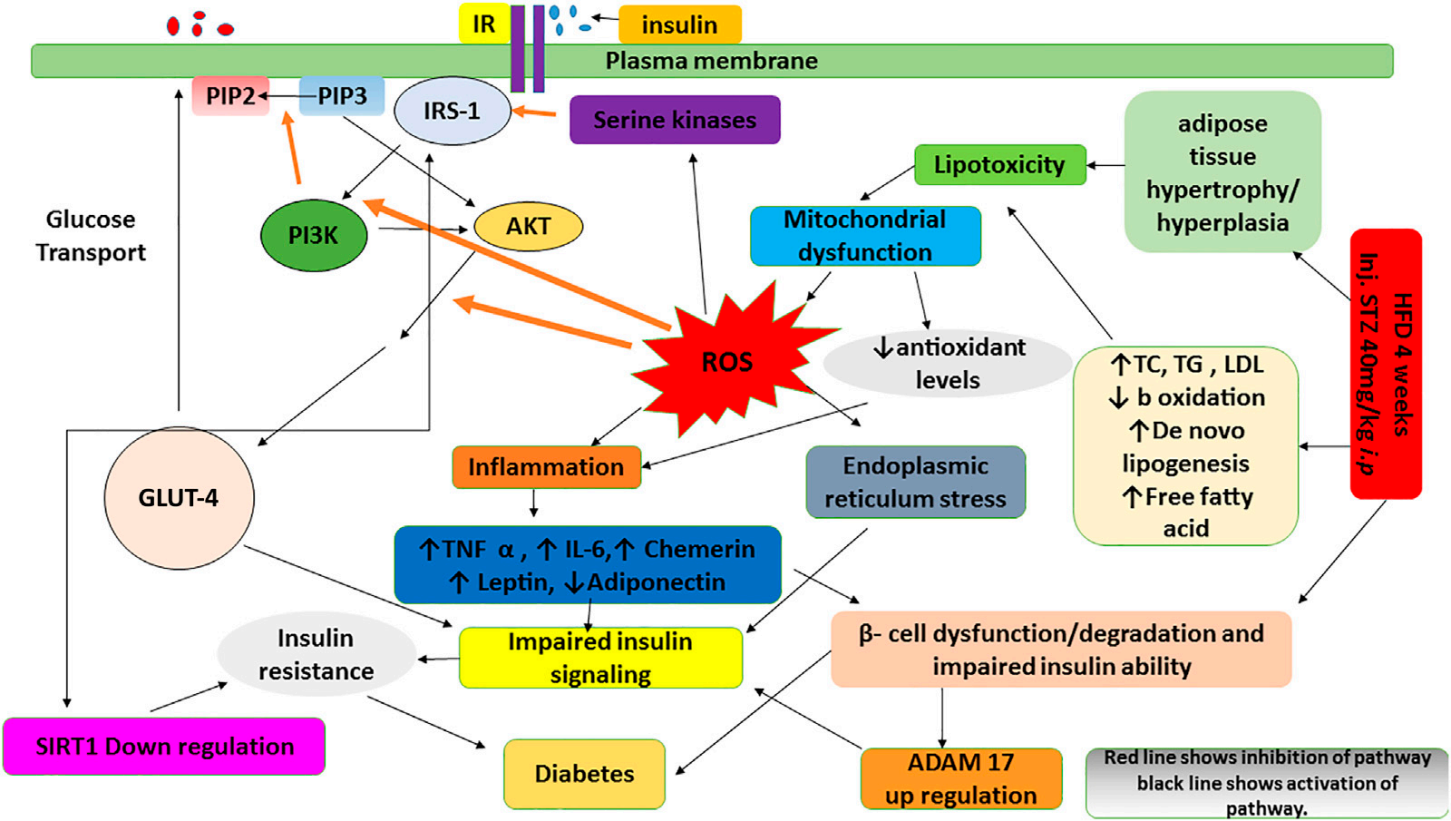
A flow diagram highlighting the effect of administration of high fat diet and streptozotocin on the production of reactive oxygen species and the cross talk of insulin signaling pathway (IRS-1, GLUT4, SIRT1 and ADAM 17) with PI3K/AKT pathway.

Administration of HFD/STZ causes activation PI3K/AKT pathway through production of reactive oxygen species (ROS). ROS and PI3K/AKT pathway is indirectly involved in mediation of insulin signaling *via* IRS-1, GLUT-4, SIRT 1 and ADAM 17. The observed antidiabetic effects of *B. brandisiana* and berbamine were found in line with the estimated expression of mRNA of IRS-1, GLUT-4, SIRT 1 and ADAM 17 as elaborated in Figure 9.

IRS-1 plays important role in the signal transduction pathway stimulated by insulin and connecting receptor of insulin to its ultimate biological actions through intermediate effectors. IRS-1 regulation differs in the liver as it has been observed in diabetic animals, which may result in distinctive changes in insulin levels in liver tissue and contribute to insulin resistance in liver. mRNA expression of IRS-1 was found lower in the livers of HFD/STZ-induced diabetic rats while the treatment of AMEBB and Berb upregulated mRNA expression of IRS-1, a key contributor in insulin resistance. Our finding is also in line with an earlier study (Zheng et al., 2011). ROS is known to play a part in activation of intracellular stress kinases and inhibition of IRS-1, thus potentially influencing insulin signaling and to promote *via* GLUT-4 translocation and down streaming of AKT resulting in insulin resistance, obesity and diabetes mellitus (Li et al., 2022). mRNA expression of GLUT-4 is downregulated in HFD/STZ-induced diabetic rats, indicating that insulin acts as a positive regulator of gene expression and explains the impaired glucose disposal. Administration of AMEBB and Berb upregulated mRNA expression of GLUT-4, thus offering protective potential of the treatment in insulin resistance and diabetes. SIRT1 controls insulin secretion by preserving pancreatic β-cells, improves insulin resistance, inflammation, mitochondrial function, controls oxidation of fatty acid and regulates hepatic glucose production. Therefore, for the treatment of insulin resistance and diabetes, SIRT1 is a favorable pharmacological target (Kitada and Koya, 2013). In our study, mRNA expression of SIRT 1 in hepatic tissues was found upregulated in HFD/STZ–exposed rats, while treatment showed is downregulation, hence supporting its protective potential against high fat diet-induced hepatic steatosis (Li et al., 2011), insulin sensitivity and oxidative stress possibly mediated through AKT pathway. These findings are also supported by earlier study of Abedimanesh et al. (2022). Similarly, medicinally active plants containing flavonoids are known to reduce SIRT 1 expression in hepatocytes (Sung et al., 2015). Diabetes is known to cause an increase in ADAM 17 overexpression due to a decrease in antioxidants and/or rise in ROS. It is reported that TNF-α has been linked to signaling pathway of insulin impairment by increasing serine phosphorylation of IRS-1, which inhibits activity of tyrosine kinase resulting in impaired downstream signaling and development of insulin resistance. Administration of AMEBB and Berb caused downregulation of mRNA expression of ADAM17 in hepatic tissues, thus offering it potential utility for the treatment of diabetes. These findings are also in line with earlier findings on another medical plant (Matthews et al., 2021). In current study, the decreased levels of mRNA of IRS-1, GLUT-4, SIRT1 and increased levels of mRNA of ADAM17 in diabetic animals, while treatment groups showed increased levels of mRNA of IRS-1, GLUT-4, SIRT1 and decreased level of mRNA of ADAM17. This helps to correlate that observed efficacy of test materials might have been achieved because of the improved insulin action which is an outcome of insulin signaling ultimately through modulation of mRNA levels of IRS-1, GLUT-4, SIRT1 and ADAM17. Our findings are also in line with earlier studies (Balbaa et al., 2016).

## Conclusion

This study demonstrates that *B. brandisiana* and Berb possess antidiabetic effects possibly mediated through attenuation of oxidative stress, glucose metabolism, inflammatory biomarkers and adipocytokines levels. Further the downregulation of IRS-1, SIRT1 and GLUT-4 and upregulation of ADAM 17 demonstrates its potential impact on glucose homeostasis, insulin resistance and chronic inflammatory markers. Thus, this study provides scientific basis to the medicinal use of *B. brandisiana* and berbamine in diabetes.

## Innovation

• It is the pioneer study showing the anti-diabetic potential of *B. brandisiana* Ahrendt in HFD and STZ-fed animals.
• Quantitative expression of mRNA of diabetic candidate genes like, IRS-1, SIRT 1, GLUT-4 and ADAM17 were studied for their role on the part of protective potential of AMEBB and Berb in diabetes.
• AMEBB and Berb demonstrated anti-inflammatory and antioxidant potential, thus providing additional support to the anti-diabetic effects of AMEBB and Berb.

## Data Availability

The original contributions presented in the study are included in the article/supplementary materials, further inquiries can be directed to the corresponding author.

## Abbreviations

AMEBB, Aqueous methanolic extract of *Berberis brandisiana*; Berb, berbamine; AST, aspartate aminotransferase; ALT, alanine transaminase; RFTs, renal function tests; SOD, superoxide dismutase; CAT, catalase; MDA, melondialdehyde; HDL, high-density lipoprotein; ROS, reactive oxygen species; ELISA, enzyme-linked immunosorbent assay; HFD, high fat diet; STZ, Streptozotocin; HPLC; highperformance liquid chromatography; LDL, Low-density lipoprotein; TC, total cholesterol; TGs, Total triglycerides; TNF- α, tumor necrosis factor alpha; IL-6, interleukin −6; IR, insulin receptor; IRS-1, insulin receptor substrate −1; GLUT-4, glucose transporter −4; SIRT 1, Sirtuin- 1; ADAM 17, A disintigrin and A metalloproteinase 17; PI3K, phosphatidylinositol 3-kinase.
